# Tetrandrine may treat membranous glomerulopathy via P13K/Akt signaling pathway regulation: therapeutic mechanism validation using Heymann nephritis rat model

**DOI:** 10.1080/21655979.2021.1973862

**Published:** 2021-09-14

**Authors:** Jiazhen Yin, Jiazhen Lin, Jin Yu, Xia Wei, Bin Zhu, Caifeng Zhu

**Affiliations:** a Department of Nephrology, Hangzhou Tcm Hospital Affiliated of Zhejiang Chinese Medical University, Hangzhou, Zhejiang, China; b School of Clinical Medicine, Zhejiang Chinese Medical University, Hangzhou, Zhejiang, China; c Department of Gastroenterology, Hangzhou Tcm Hospital Affiliated of Zhejiang Chinese Medical University, Hangzhou, Zhejiang, China

**Keywords:** Tetrandrine, membranous glomerulopathy, network pharmacology, molecular mechanism

## Abstract

Membranous glomerulopathy (MGN) is an autoimmune kidney disease that is the primary cause of nephrotic syndrome (NS) in adults. Tetrandrine, a bisbenzylisoquinoline alkaloid, is known to have numerous pharmacological effects. In this study, network pharmacology analysis and experimental validation were conducted to analyze the mechanisms by which tetrandrine functions as a therapeutic intervention for MGN. A systematic network pharmacology method was applied to identify potential targets and determine the therapeutic mechanism of tetrandrine in MGN treatment. A Heymann nephritis (HN) rat model was developed to assess the therapeutic effects of tetrandrine on NS and validate the predicted molecular mechanisms. We obtained 86 potential targets of tetrandrine for the treatment of NS. *In vivo* experiments showed that tetrandrine could reduce the 24-h urine protein content, decrease glomerular basement membrane proliferation, and significantly decrease thylakoid stroma and cell proliferation in the HN rat kidney tissue. Moreover, tetrandrine suppressed kidney cell apoptosis and upregulated the expression of nephrin and podocin in HN model rats. qRT-PCR results revealed that tetrandrine inhibited IL-1β, TNFα, and MCP-1 levels in HN model rats. Western blot results indicated that tetrandrine can protect against MGN via the PI3K/Akt signaling pathway. Thus, by using a combination of network and experimental pharmacology methods, we demonstrate that tetrandrine can treat MGN via the PI3K/Akt signaling pathway and provide novel insights into the mechanisms underlying tetrandrine-mediated management of MGN.

## Graphical abstract

**Figure:**
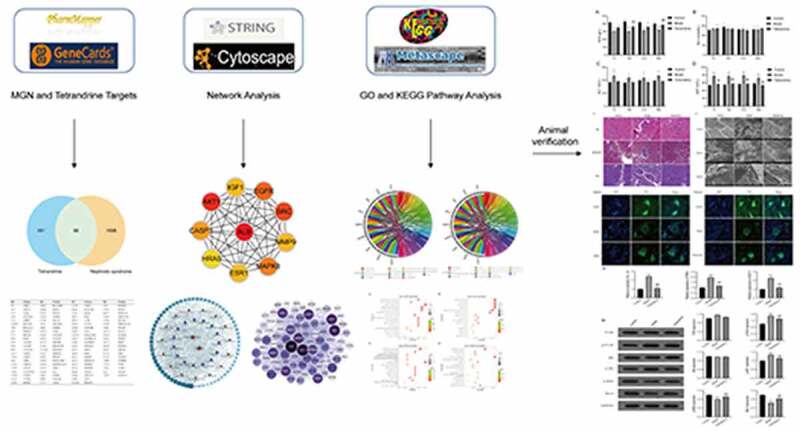

## 1. Introduction

Membranous glomerulopathy (MGN), also known as membranous nephropathy (MN), is a slow-progressing immune and inflammation–associated disease with a high prevalence in the adult population, regardless of ethnicity or age group, and is more widely reported in men than women [1,2]. MGN is characterized by the deposition of immune complexes consisting of IgGs (mainly IgG1 and IgG4) and C5b‐9 under the glomerular epithelium, thickening of the glomerular basement membrane (GBM), and effacement of the podocyte foot processes. Clinical manifestations include proteinuria or nephrotic syndrome (NS) [3,4].

A large-scale, nationwide biopsy study including more than 70,000 cases in China revealed a significant upward trend in the frequency of MN incidence over the past decade, with an average annual growth rate of 13% [5], which inevitably leads to increasing financial burdens on health care systems and families. The etiology of MGN remains unclear, and the prognosis is diverse and variable, with approximately 1/3 of patients progressing to end-stage renal disease (ESRD) within 5–15 years [3,6]. Clinical treatment options for MGN include general symptomatic support and the administration of glucocorticoids, immunosuppressants, or glucocorticoids in combination with immunosuppressants [7]. Wu CC et al. demonstrated that immunosuppressants in combination with glucocorticoids significantly improved remission rates and reduced the risk of progression to ESRD or death compared to other treatments for MGN [8]. Certain advantages have been reported with the use of traditional Chinese medicine (TCM) for MGN treatment, especially for reducing proteinuria, managing the side effects of immunosuppressive drugs, and mitigating hypercoagulable or gastrointestinal mucosal edema complications [9,10].

Tetrandrine, a bisbenzylisoquinoline alkaloid, is the main active component isolated from the Chinese herbal radix *Stephania tetrandra* and has been reported to exhibit a variety of pharmacological effects. It is usually used in clinical practice to treat arthritis, silicosis, and hypertension; manage tumor growth; treat liver fibrosis; and protect liver cells [11]. Moreover, powdered tetrandrine is a highly active component in Fangqi Huangqi Tang, a well-known traditional Chinese medicine prescribed for the treatment of NS, that has been shown to exert anti-inflammatory effects against edema and swelling in rat paws [12–14]. To our knowledge, no prior studies have reported the effect of tetrandrine in MGN treatment or elucidated its therapeutic mechanism in this application.

Network pharmacology, first introduced by British pharmacologist Hopkins, has proved to be an effective strategy for revealing the complicated relationship between TCMs and disease [15,16]. TCMs are considered to exert their therapeutic functions through a multi compound–target–pathway network; hence, the molecular mechanisms of therapeutic agents can be clarified by network pharmacology in a systematic manner [17]. In this study, we applied the network pharmacology method to analyze the potential molecular mechanism of tetrandrine in the treatment of MGN and created a Heymann nephritis (HN) rat model to verify the results. We hypothesized that tetrandrine may inhibit the development of MGN via certain pathway regulation.

## 2. Materials and methods

### 2.1. Therapeutic targets for NS

Potential therapeutic targets for NS were collected from Gene Cards (https://www.genecards.org/) [18] using the key word ‘nephrotic syndrome’ (2020.12.9), which yielded a large number of results related to genomes, transcriptomes, proteomes, and genetics as well as clinical and functional information from 150 web sources.

### 2.2. Screening of bioactive components and target proteins

The bioactive components and targets of tetrandrine were obtained from the PharmMapper database (http://lilab.ecust.edu.cn/pharmmapper/) [19]. The gene module in the NCBI database was used to standardize the biological target information and establish a chemical composition target database.

### 2.3. PPI network construction

The primary targets of tetrandrine for NS treatment were identified using a Venn diagram [20], and protein–protein interaction (PPI) data were obtained from the STRING database (https://string-db.org/) [21], which is typically used to collect known protein interactions.

### 2.4. GO and KEGG pathway enrichment analysis

Gene ontology (GO) and Kyoto encyclopedia of genes and genome (KEGG) pathway enrichment analyses were performed using the respective public databases [GO: http://geneontology.org/ and KEGG: https://www.genome.jp/kegg/]. GO enrichment analysis includes the following key aspects: biological process (BP), molecular function (MF), and cellular components (CCs).

The hypergeometric distribution model was used to evaluate whether the target gene set was significantly associated with the specific gene ontology or biological pathway. The calculation formula is as follows:

$$P=1\sum_{i=0}^{k-1}\frac{\left(\begin{array}{c} M\\ i \end{array}\right)\left(\begin{array}{c} N-M\\ n-i \end{array}\right)}{\left(\begin{array}{c} N\\ n \end{array}\right)}$$

where N represents the total number of genes from the reference terms, M is the number of genes annotated to specific GO terms or pathways, n is the number of identified tetrandrine-target genes, and k is the number of the common genes between tetrandrine-target genes and the reference set. The clusterProfiler package in R (4.0) (http://www.bioconductor.org/packages/release/bioc/html/clusterProfiler.html) was adopted to perform the GO and KEGG pathway enrichment analyses with p-values < 0.01 adjusted by the Bonferroni correction.

### 2.5. Constructing a component–target–disease network

The Cytoscape software [22] was used to construct a component–target–disease network. The core targets were screened from the cytoscapehub plugin to form a core network to assess the relationship of the KEGG-associated entries in compound diseases.

### 2.6. Construction of the HN rat model *[23]*

Adult male Sprague–Dawley (SD) rats (6–7 weeks old, 180–220 g weight) were purchased from Shanghai SLAC Laboratory Animal Co., Ltd. (Shanghai, China). All rats were housed under standard specific pathogen–free conditions with a 12-h light/dark cycle at 22–24°C and allowed to eat a standard diet and drink ad libitum. Following one-week, male SD rats was divided into three groups (n = 12): control, model, and tetrandrine. The rats were injected with Fx1A antiserum (8 mg/kg, Probetex, USA) in the tail vein in all groups except the control group, which was injected with an equal amount of saline. After 10 d, 24-h urine samples were collected separately from all rats in metabolic cages to measure urine protein (UP) levels. Rats with 24-h urine protein (24 UP) levels below 10 mg were selected from the normal control group, and rats with 24 UP levels above 20 mg from the remaining two groups were used in the follow-up experiments. The treatment group was given 20 mg/kg/d of tetrandrine (C_38_H_42_N_2_O_6_, HPLC≥98%, Aladdin 877,811, chemical structure is shown in Figure 8(a).) for 4 weeks by gavage, and the control and HN model groups were given equal amounts of distilled water. After 7, 14, 21, and 28 days of drug treatment, urine was collected in the metabolic cage for a 24-h period. The 24-h urine was collected and measured by an Aeroset biochemical analyzer (Abbott, Chicago, IL, USA). Serum was taken for the detection of albumin (ALB), creatinine (SCr), and aminotransferase (AST and ALT) levels using a Hitachi biochemical analyzer (Hitachi 7600, Tokyo, Japan). All animal experiments were guided and approved by the Animal Care and Use Committee of Hangzhou TCM Hospital Affiliated of Zhejiang Chinese Medical University (approval number: IACUC-20190325-05).

### 2.7. Preparation and staining of pathological renal tissue sections

The kidney was excised from the coronal plane of the renal hilum. The thickness was approximately 2 mm. The tissue was fixed with 10% neutral formaldehyde and embedded in paraffin. The thickness of the section was 2 μm. HE, MASSON, and PAS staining were performed. Pathological changes in the renal tissue were observed using an optical microscope.

### 2.8. Transmission electron microscopy (TEM)

The renal tissue was cut into 1 mm^3^ pieces and fixed at 4 °C for more than 4 h with 3.75% glutaraldehyde. Then, the samples were rinsed five times with PBS, fixed in 2% osmic acid at 4 °C for 2 h, rinsed three times with distilled water, dehydrated and soaked in acetone, and embedded using a kit before slicing. The slices with thicknesses of 80 − 90 nm were placed on copper mesh, dyed in uranyl acetate dyeing solution at room temperature for 15 − 30 min, washed with double distilled water, dried by filter paper, then dyed in lead citrate solution at room temperature for 10 − 20 min. The dyed slices were washed with double distilled water and dried on filter paper prior to examination via a transmission electron microscope. The thickness of the basement membrane was also measured.

### 2.9. Immunofluorescence analysis

The sections with a thickness of 2 μm were incubated with mouse anti-rat nephrin monoclonal antibody and rabbit anti-rat podocin (1:100), respectively, at 4 °C overnight, and then rinsed thrice with PBS buffer. The sheep anti-rat and sheep anti-rabbit antibodies labeled with FITC were subjected to room temperature reaction for 45 min, respectively. Observed the seal after PBS washing.

### 2.10. Western blot analysis

Crushed kidney tissue was homogenized with lysis buffer (Beyotime Biotechnology, Shanghai, China) and centrifuged, and the supernatant was collected from the resultant samples. After quantification using BCA (bicinchoninic acid) methods (Thermo Scientific, Darmstadt, Germany), 30 µg of protein lysates were separated on 10% SDS-PAGE (sodium dodecyl sulfate-polyacrylamide gel electrophoresis) (Invitrogen) and then transferred onto PVDF (polyvinylidene fluoride) membranes (Millipore, Whatman, Germany), according to the standard protocol. After being blocked using 5% skim milk (in TBS, Beyotime), PVDF membranes were incubated in solutions containing primary antibody (1:1000) at 4°C overnight, followed by incubation with secondary horseradish peroxidase-conjugated goat anti-rabbit/mouse IgG antibody (1:1000) for 1 h at 37°C. An enhanced chemiluminescence system and Image-Pro Plus 6.0 software were used for membrane imaging. All antibodies were purchased from Boster Biotechnology.

### 2.11. Quantitative real-time PCR (qRT-PCR) analysis

Total RNA was extracted from kidney tissues using TRIzol reagent (Invitrogen, Shanghai, China). The first-strand cDNA was reverse transcribed using a Bestar qPCR RT Kit (DBI Bioscience, Ludwigshafen, Germany). One microgram of DNA sample was used for the PCR amplification of related genes under the following reaction conditions: 95°C for 4 min, 38 cycles of 94°C for 30 s, 60°C for 30 s, and 72°C for 30 s. Primers were synthesized by Sangon (Shanghai, China) and are listed in Table S1. A DBI Bestar® SybrGreen qPCR master Mix kit (DBI Bioscience) and Agilent Stratagene Mx3000P Real time PCR instrument (DBI Bioscience) were used for the amplification. The relative expression level of each gene was calculated using the 2^−ΔΔCt^ method [24]. *GAPDH* was used as the internal reference gene.

### 2.12 TUNEL assay

Apoptosis was assessed in rat kidney tissues using a TUNEL assay kit (Roche, Mannheim, Germany). Briefly, kidney tissue sections were pre-treated with 0.1% Triton X-100 (Beyotime) and incubated with TUNEL reaction mixture and propidium iodide according to the manufacturer’s instructions. Image acquisition was performed using a fluorescence microscope (Olympus, Japan, magnification × 200).

### 2.13. Statistical analysis

The data were processed using SPSS 25.0 statistical software. One-way ANOVA followed by Turkey test was applied in the analysis, where *P* < 0.05 was considered statistically significant.

## 3. Results

This study aimed to explore the alleviating effect of tetrandrine on MGN and specifically analyze the potential molecular mechanisms of tetrandrine with respect to the PI3K/Akt pathway. The pharmacological effect and potential mechanism of tetrandrine on MGN were first analyzed using a network pharmacology method. An HN rat model was then established for experimental verification. We speculate that tetrandrine may treat MGN through the regulation of the PI3K/Akt pathway.

### 3.1. Therapeutic target identification for NS

**Figure 1.**
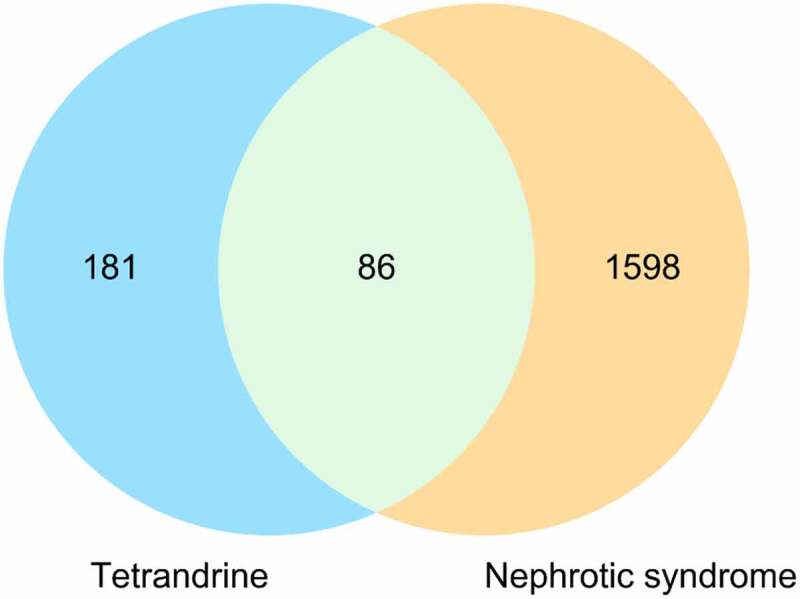
Venn diagram of targets for tetrandrine treating MGN

A total of 1,598 known therapeutic targets for NS were obtained from the Gene Card database. We downloaded 267 bioactive compound targets of tetrandrine from the PharmMapper database. Through the intersection of the drug and disease targets, we obtained 86 genes (Figure 1) that may play a major role in treating NS with tetrandrine. The details of these targets are shown in Table 1.

**Table 1.** The main targets of tetrandrine in the treatment of nephrotic syndrome (NS)

ID	Name	ID	Name	ID	Name	ID	Name
28	ABO	1991	ELANE	3718	JAK3	142	PARP1
207	AKT1	2099	ESR1	8850	KAT2B	5105	PCK1
213	ALB	2224	FDPS	3791	KDR	8654	PDE5A
308	ANXA5	2539	G6PD	3815	KIT	5294	PIK3CG
353	APRT	2739	GLO1	3932	LCK	5328	PLAU
23,621	BACE1	2811	GP1BA	3934	LCN2	5566	PRKACA
598	BCL2L1	2885	GRB2	4048	LTA4H	5660	PSAP
635	BHMT	2936	GSR	4069	LYZ	5879	RAC1
673	BRAF	2944	GSTM1	11,253	MAN1B1	5916	RARG
695	BTK	2950	GSTP1	1432	MAPK14	5950	RBP4
834	CASP1	3074	HEXB	5599	MAPK8	5972	REN
836	CASP3	3156	HMGCR	4193	MDM2	6256	RXRA
890	CCNA2	27,306	HPGDS	4233	MET	6401	SELE
1017	CDK2	3251	HPRT1	4282	MIF	6714	SRC
1022	CDK7	3265	HRAS	4311	MME	6772	STAT1
629	CFB	3290	HSD11B1	4321	MMP12	7068	THRB
1118	CHIT1	3320	HSP90AA1	4314	MMP3	7276	TTR
1384	CRAT	3479	IGF1	4318	MMP9	7421	VDR
1495	CTNNA1	3558	IL2	4846	NOS3	331	XIAP
1508	CTSB	3643	INSR	2908	NR3C1	7535	ZAP70
1513	CTSK	3683	ITGAL	5009	OTC		
1956	EGFR	3717	JAK2	23,569	PADI4

### 3.2. Construction and analysis of the PPI network of the target proteins

**Figure 2.**
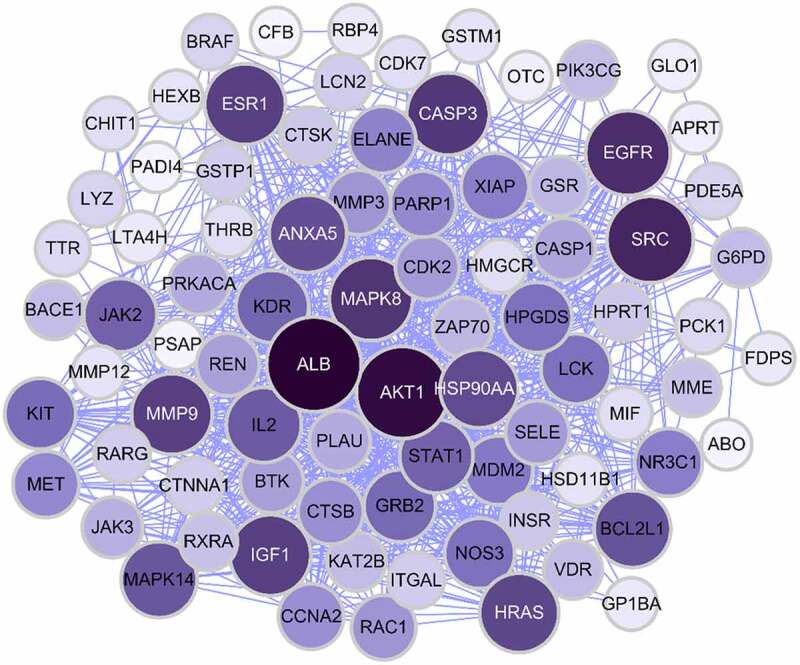
The nerwork of MGN-related targets

To determine how the overlapping genes interact, we uploaded the relevant information to the STRING (Search Tool for the Retrieval of Interacting Genes) database and subsequently constructed a PPI network, which contained 86 nodes and 737 edges (Figure 2); in the figure, the larger sizes and darker colors of the target nodes indicate a greater degree of the nodes in the PPI network. The PPI network results revealed a complex relationship between the investigated genes.

### 3.3. GO and KEGG pathway enrichment analysis

**Figure 3.**
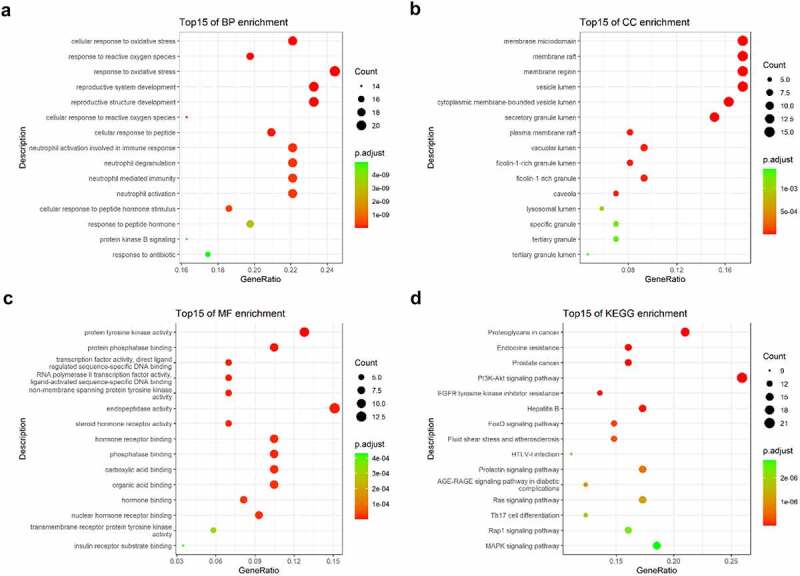
GO and KEGG enrichment analysis. (a) The top 15 significantly enriched terms in biological process (BP); (b) The top 15 significantly enriched terms in cellular component (CC); (c) The top 15 significantly enriched terms in molecular function (MF); (d) The top 15 significantly enriched terms in KEGG pathway. Gene ratio = count/set size

The intersected targets of tetrandrine and NS were analyzed using R. GO annotation showed that the targets of PPI network were classified into 917 BP terms, primarily associated with cellular response to oxidative stress, response to oxidative stress, reproductive system development, reproductive structure development (Figure 3(a)); 31 CC terms, mainly in membrane microdomain, membrane raft, membrane region, vesicle lumen (Figure 3(b)); and 43 MF terms, mainly involved in protein tyrosine kinase activity and endopeptidase activity (Figure 3(c)) (*P* < 0.01). KEGG pathway analysis revealed that the targets of the PPI network were assigned to 134 KEGG pathways based on *P* < 0.01. As shown in Figure 3(d), KEGG pathway enrichment analysis suggested that the targets were mainly associated with the signaling pathway of proteoglycans in cancer and the PI3K-Akt signaling pathway. The detailed results are shown in Table 2.

**Table 2.** The top 15 KEGG pathways of tetrandrine related target genes

ID	Description	p.adjust	Count
hsa05215	Prostate cancer	9.61E-10	13
hsa01522	Endocrine resistance	9.61E-10	13
hsa05205	Proteoglycans in cancer	9.61E-10	17
hsa04151	PI3K-Akt signaling pathway	1.24E-09	21
hsa01521	EGFR tyrosine kinase inhibitor resistance	1.29E-08	11
hsa05161	Hepatitis B	2.22E-08	14
hsa04068	FoxO signaling pathway	1.87E-07	12
hsa05418	Fluid shear stress and atherosclerosis	3.23E-07	12
hsa04917	Prolactin signaling pathway	6.75E-07	9
hsa05166	HTLV-I infection	6.75E-07	14
hsa04933	AGE-RAGE signaling pathway in diabetic complications	1.06E-06	10
hsa04014	Ras signaling pathway	1.17E-06	14
hsa04659	Th17 cell differentiation	1.72E-06	10
hsa04015	Rap1 signaling pathway	2.33E-06	13
hsa04010	MAPK signaling pathway	2.73E-06	15

### 3.4. Enrichment analysis of hub genes

**Figure 4.**
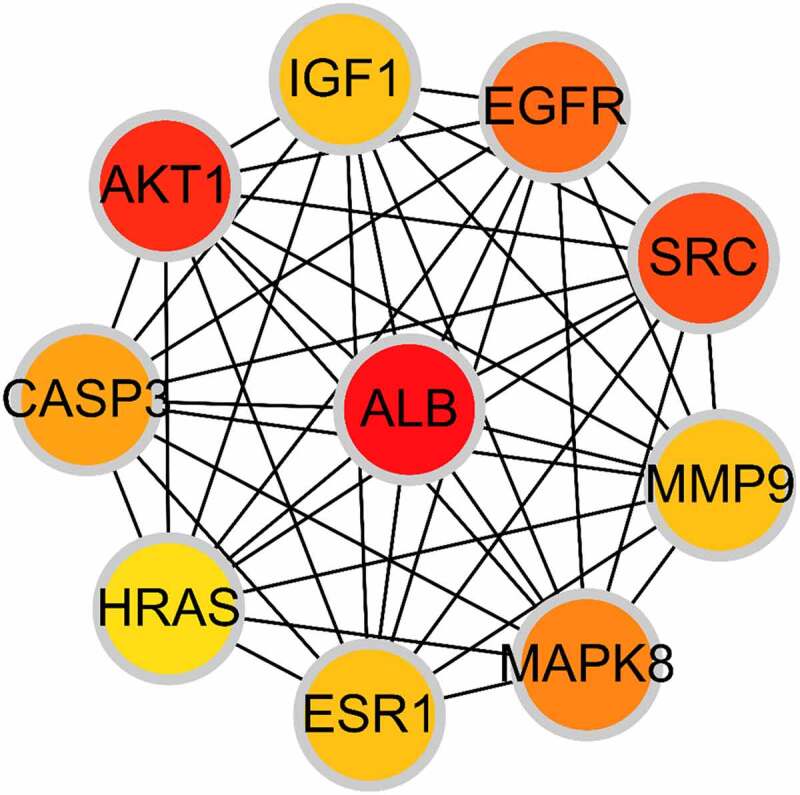
The PPI network of 10 hub genes. IGF1: Insulin-like growth factor 1; AKT1: AKT Serine/Threonine Kinase 1; CASP3: Caspase 3; HRAS: HRas Proto-Oncogene, GTPase; ESR1: Estrogen Receptor 1; MAPK8: Mitogen-Activated Protein Kinase 8; MMP9: Matrix Metallopeptidase 9; SRC: SRC Proto-Oncogene, Non-Receptor tyrosine kinase; EGFR: Epidermal Growth Factor Receptor; ALB: Albumin

**Figure 5.**
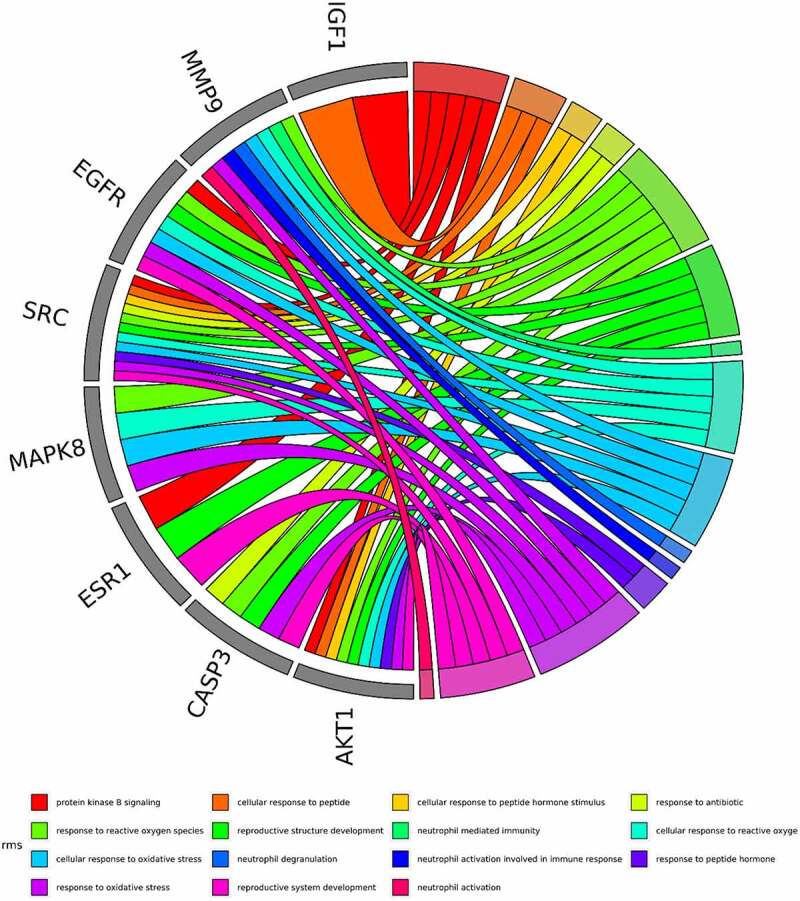
The top 15 GO terms of hub genes

**Figure 6.**
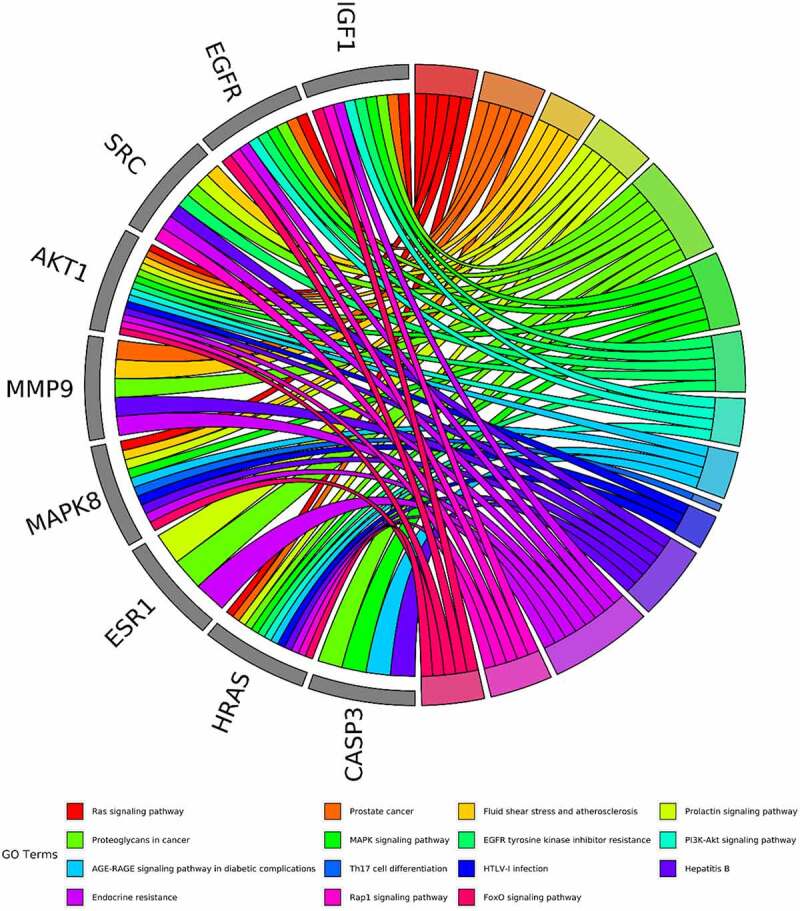
The top 15 pathways of hub genes

Based on the core-target PPI network, hub genes were selected using cytoHubba, which revealed IGF1, AKT1, CASP3, HRAS, ESR1, MAPK8, MMP9, SRC, EGFR, and ALB as the major hub nodes in the core-target PPI network. The PPI network of the hub genes is illustrated in Figure 4. The correlation analysis between the core-target and the top 15 most representative GO terms is shown in Figure 5, and the correlation analysis between the core-target and the top 15 signaling pathways is shown in Figure 6.

**Figure 7.**
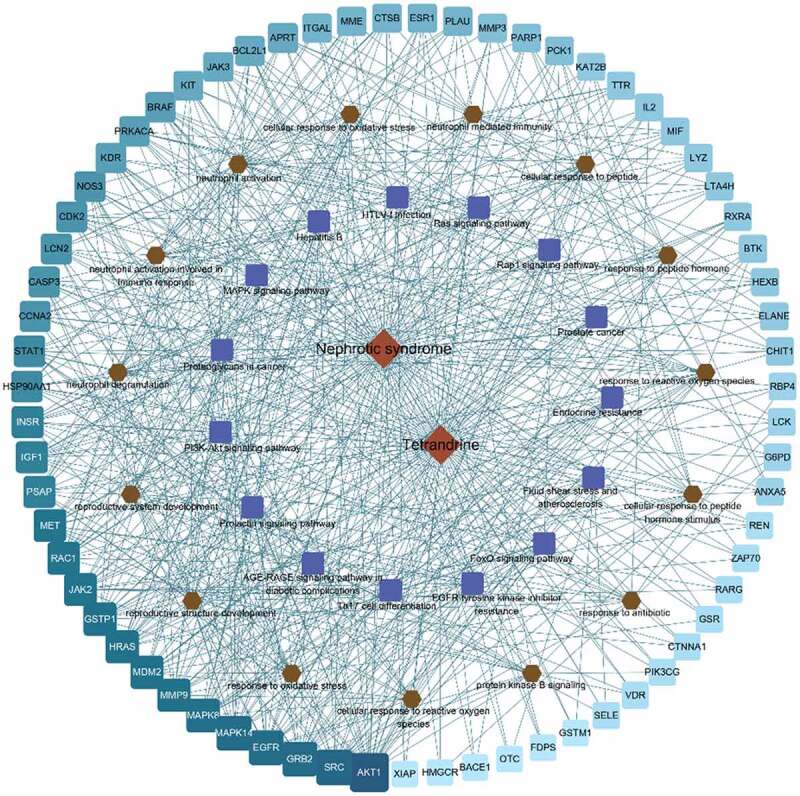
Compound action disease targets were associated with the top 15 KEGG pathways and the top 15 GO terms

To further elucidate the therapeutic potential of tetrandrine in NS, we constructed a network between the 86 disease targets and the top 15 signaling pathways of the top 15 BP terms (Figure 7). In the Figure 7, light blue to cyan squares indicated the disease targets; the closer the cyan square is, the higher the correlation is.

### 3.5. 24 UP content and kidney tissue assays

**Figure 8.**
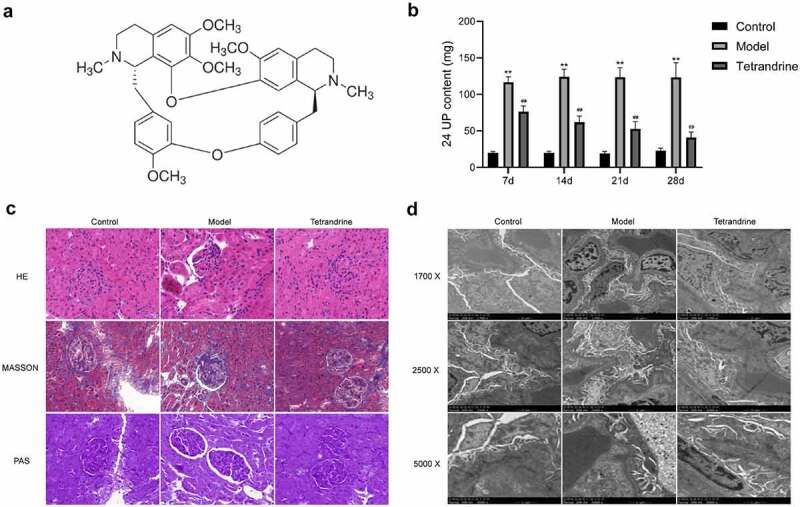
(a) The chemical structure of tetrandrine. (b) The 24 UP contents were detected after 7, 14, 21 and 28 days of tetrandrine treat in control, HN model and tetrandrine groups. (c) The representative images of the histology staining on the kidney tissue. (d) The representative images of TEM on the kidney tissue. **, *P* < 0.01 vs. control group; ##, *P* < 0.01 vs. model group

To verify the therapeutic effect of tetrandrine on MGN, we established a rat model of HN, which is a widely used model for evaluating MGN. We detected the 24 UP content of nephritic rats at 7, 14, 21 and 28 days after treatment. The results showed that the 24 UP content of the HN model group was significantly higher than that of the control group; compared with the HN model group, the 24 UP content of the tetrandrine intervention group was significantly lower, and gradually decreased with the extension of the intervention time (Figure 8(b)).

To visualize the efficacy of tetrandrine for the treatment of MGN, we obtained kidney sections of SD rats and performed hematoxylin–eosin, Masson, and periodic acid–Schiff staining (Figure 8(c)). Compared with the control group, the kidney tissues of the rats in the HN model group showed enlarged glomeruli, diffuse thickening of GBM, and increased thylakoid stroma and thylakoid cell proliferation. Decreased GBM proliferation and significantly decreased thylakoid stroma and cell proliferation were observed in the kidney tissues of rats treated with tetrandrine.

In addition, the TEM results demonstrate that compared with the control group, the HN model group showed a large amount of immune complex deposits under the epithelial cells and an extensive fusion or disappearance of podocyte peduncles in the kidney tissue. Compared with the HN model group, the tetrandrine intervention group showed less immune complex deposits under the epithelial cells and a significantly less fusion of podocyte peduncles (Figure 8(d)).

These results indicate that tetrandrine was able to significantly mitigate the effects of MGN.

### 3.6 Tetrandrine regulates ALB, SCr, AST, ALT levels in HN rats

**Figure 9.**
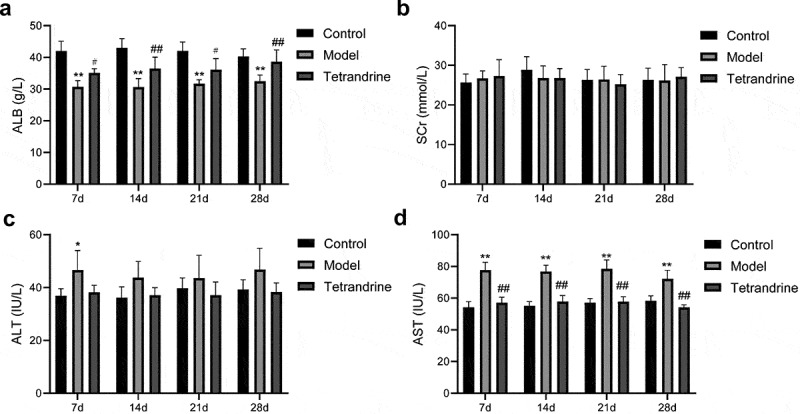
After 7, 14, 21 and 28 days of tetrandrine treat, the serum content of ALB (a), SCr (b), ALT (c) and AST (d) level in control, model and tetrandrine groups. *, *P* < 0.05 and **, *P* < 0.01 vs. control group; #, *P* < 0.05 and ##, *P* < 0.01 vs. model group

As shown in Figure 9, after 7, 14, 21 and 28 days, the HN model group exhibited significantly reduced ALB levels and increased AST levels compared with the control group. After 7 days, the ALT level was significantly increased, and no further significant differences were observed after 14, 21 and 28 days. Compared with the HN model group, tetrandrine treatment for 7, 14, 21 and 28 days caused a significant increase in the ALB level and a significant decrease in the AST level. The ALT level was significantly lower after 7 and 28 days of tetrandrine treatment, and no further significant differences were observed after 14 and 21 days of treatment. There was no significant difference in the SCr levels among the three groups.

### 3.7 Anti-oxidative effect of tetrandrine in HN rats

Oxidative stress has been implicated in inflammation, which is directly related to the level of malondialdehyde (MDA) and the activities of superoxide dismutase (SOD) and glutathione (GSH). Overproduction of MDA and low activities of SOD and GSH-Px were observed in the sera and kidneys of HN rats, which could be reversed after treatment with tetrandrine (Table 3).

**Table 3.** The anti-oxidative effect of tetrandrine in Heymann nephritis (HN) rats

		Control	Model	Tetrandrine
Kidney	SOD (U/mL)	145.67 ± 9.03	78.55 ± 9.91**	113.63 ± 5.52 ^##^
	MDA (nmol/mL)	8.16 ± 0.34	12.46 ± 0.58**	9.73 ± 0.42 ^##^
	GSH-px (μmol/L)	5521.40 ± 505.74	2861.92 ± 557.51**	4408.34 ± 378.48 ^#^
Serum	SOD (U/mL)	356.94 ± 30.35	216.42 ± 24.96**	281.79 ± 17.10 ^#^
	MDA (nmol/mL)	8.20 ± 0.49	16.36 ± 0.87**	11.49 ± 0.76 ^##^
	GSH-px (μmol/L)	543.55 ± 21.82	257.49 ± 28.20**	418.39 ± 35.31^##^

The levels of MDA and the activities of SOD and GSH-Px in serum and kidney of HN and control rats were detected after 4-week administration of tetrandrine. ** *P* < 0.01 vs. control rats; # *P* < 0.05 and ## *P* < 0.01 vs. HN rats.

### 3.8 Tetrandrine suppresses renal cell apoptosis

**Figure 10.**
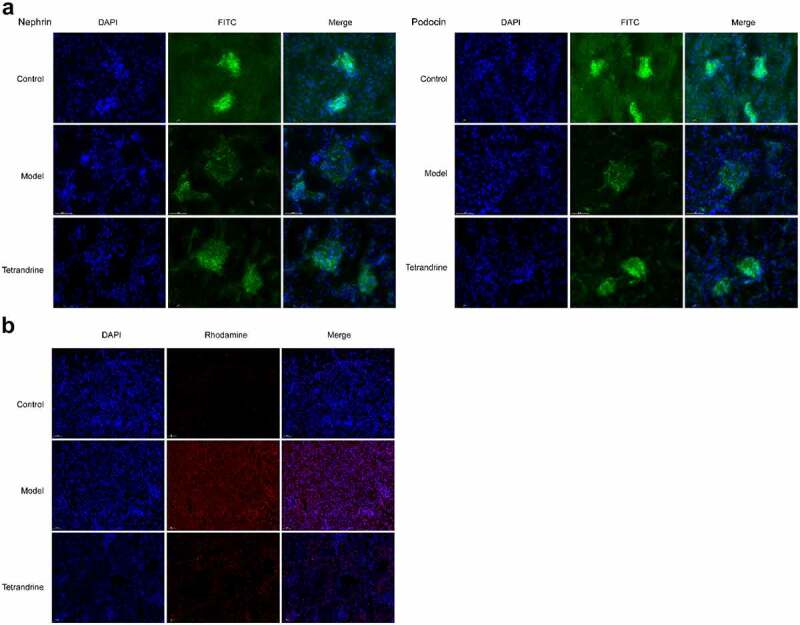
(a) Nephrin and podocin expression of kidney tissues were detected by immunofluorescence staining. (b) The kidney tissues cell apoptosis was analyzed via TUNEL

The development of kidney disease is related to podocytes; hence, we examined the expression of two podocyte molecules, namely nephrin and podocin, using immunofluorescence techniques. The results showed that the number of histiocytes was reduced in the kidney tissue of the HN model rats compared with the control group, and the expression of nephrin and podocin was significantly reduced, and their distribution was abnormal. The distribution characteristics of nephrin and podocin in the glomeruli of HN rats changed from continuous linear distribution to diffuse granular distribution; compared with the HN model group, the number of histiocytes in the kidney tissue of the rats treated with tetrandrine increased, and the expression of nephrin and podocin increased significantly, changing from a diffuse granular distribution to a uniform continuous linear distribution along the capillary collaterals (Figure 10(a)). In addition, the TUNEL assay revealed the ability of tetrandrine to inhibit apoptosis in rat kidney tissues (Figure 10(b)).

### 3.9 Tetrandrine improves kidney injury via PI3K/Akt in HN rats

**Figure 11.**
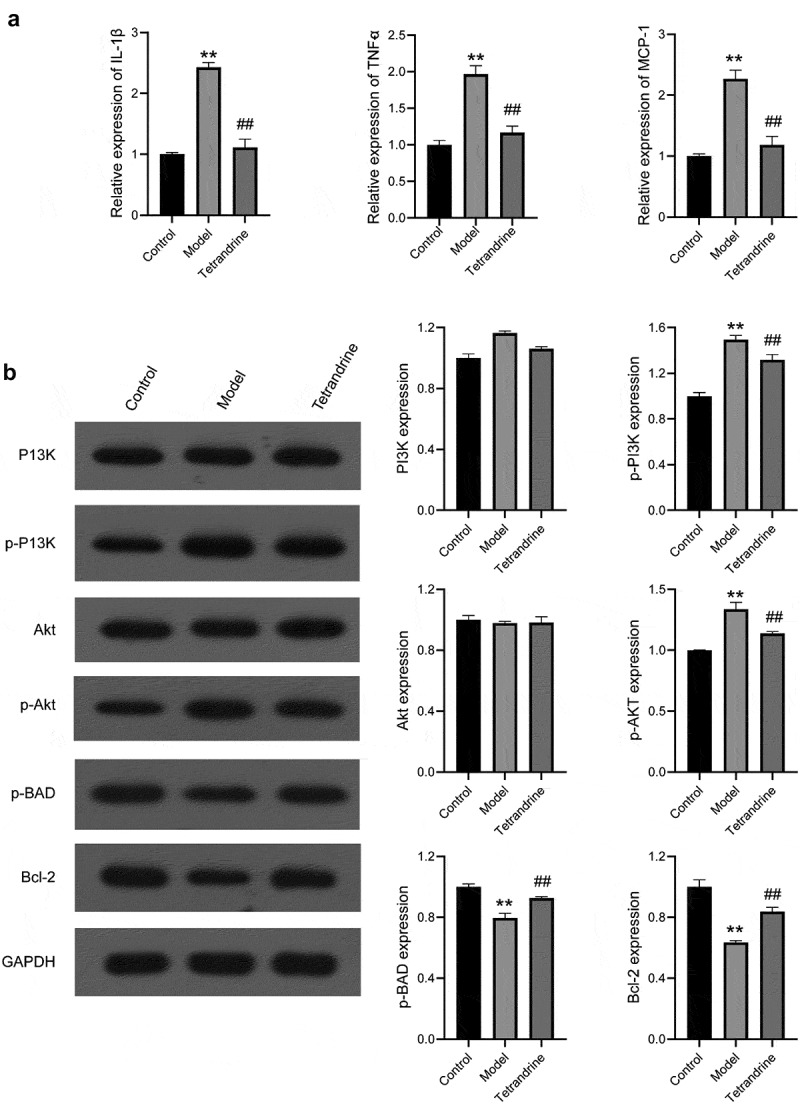
(a) The expression levels of IL-6, TNFα and MCP-1 in the kidney tissues were examined by qRT-PCR in control, model and tetrandrine groups rats. (b) The expression of p-PI3K, PI3K, Akt, p-Akt, p-BAD, Bcl-2 were examined by western blot in control, model and tetrandrine groups rats. **, *P* < 0.01 vs. control group; ##, *P* < 0.01 vs. model group

Compared with the results of the control group, higher expression of IL-6, TNFα, and MCP-1 was observed in the kidney tissues of the rats in the HN model group; compared with the results of the HN model group, lower expression of IL-6, TNFα, and MCP-1 was observed in the kidney tissues of the rats in the tetrandrine group (Figure 11(a)).

As shown in Figure 11(b), the protein contents of p-PI3K and p-Akt in the kidney tissues of the rats in the HN model group were significantly increased compared with those in the rats of the control group, and the protein contents of p-PI3K and p-AKT in the kidney tissues of the rats in the tetrandrine group were significantly decreased compared with those in the rats of the HN model group. The protein contents of p-BAD and Bcl-2 in the kidney tissues of the rats in the HN model group were significantly decreased compared with those in the rats of the control group, and the protein contents of p-BAD and Bcl-2 in the kidney tissues of the rats in the tetrandrine group were significantly increased compared with those in the rats of the HN model group. No difference was observed with respect to the protein contents of PI3K and Akt.

## 4. Discussion

MGN is an organ-specific autoimmune disease and a major cause of mortality in patients with NS worldwide [25]. Despite the extensive use of immunosuppressants and corticosteroids for inducing disease remission and reducing the risk of progression to ESRD or death [6,26–28], as many as 20% of the patients with MN are refractory to treatment [29] and up to 40% develop ESRD during the course of treatment [30]. In addition, immunosuppressive agents are associated with significant toxicity, particularly infections, malignancy, and infertility [31,32]. Therefore, further research into the discovery or development of more effective drugs for the treatment of MGN is needed. In this study, we applied network pharmacology to reveal the targets and mechanisms of action of tetrandrine in the treatment of NS. Meanwhile, we established an HN rat model and experimentally verified the ability of tetrandrine to treat NS through PI3K/Akt signaling pathway.

In this research, by intersecting the active ingredient targets of tetrandrine and the targets of NS treatment, we obtained 86 target genes. The results of the network analysis showed that the occurrence of NS is closely related to the cellular response to oxidative stress, neutrophil activation involved in immune response. Oxidative stress is a major mediator of tissue and cell injuries, and numerous studies have shown that the inhibition of oxidative stress is beneficial for maintaining kidney health [33–35]. Elvin found that adrenocorticotropic hormone and a selective agonist for the melanocortin 1 receptor (MC1R) exert beneficial actions in experimental MN with reduced proteinuria, reduced oxidative stress, and improved glomerular morphology and function [33]. In addition, it has also been demonstrated that the serum of patients with NS reveals increased levels of oxidative stress [36,37]. In our study, tetrandrine treatment significantly reduced MDA production and increased SOD and GSH-px expression levels. These results indicate that tetrandrine may protect the kidneys by inhibiting oxidative stress.

The suppressive effect of tetrandrine on MGN might also be attributable to its ability to inhibit the expression of some pro-inflammatory cytokines. IL-1β, IL-6, and TNFα are important cytokines that serve as essential mediators of the immune response and inflammatory reactions in patients with chronic renal failure [38]. In the present study, using qRT-PCR analysis, we demonstrated that the expression of IL-1β, TNFα, and MCP-1 was significantly increased in the HN model group and significantly decreased in the tetrandrine group. Tetrandrine may be exhibiting its anti-inflammatory effect and the subsequent renoprotection through the inhibition of IL-1β, TNFα, and MCP-1.

It has been shown that MGN is a glomerular disease characterized by proteinuria, which is mainly caused by podocyte dysfunction and depletion [39,40]. Several molecules synthesized by podocytes, such as podocin and nephrin [41,42], play key roles in maintaining the integrity of the glomerular filtration barrier. It has also been shown that tetrandrine can alleviate transient receptor potential cation channel protein 6 (TRPC6) expression-induced podocyte injury [43]. In the present study, low podocyte biomarker expression (nephrin and podocin) and high 24 UP content were observed in the HN model group, indicating that tetrandrine treatment significantly inhibited nephrin and podocin expression and decreased the content of 24 UP. These results suggest that the protective effect of tetrandrine on podocytes may partially contribute to its albuminuria-mitigating activity.

To further validate the mechanism by which tetrandrine functions in MGN treatment, we performed KEGG enrichment analysis, the results of which indicated that tetrandrine treatment of MGN was mainly related to the PI3K/Akt signaling pathway. The PI3K/Akt pathway was found to be involved in cell proliferation, differentiation, metabolism, and apoptosis and may also be involved in podocyte injury [44], mesangial cell hypertrophy [45], and epithelial-mesenchymal transition of renal proximal tubular cells [46]. Additionally, Li et al. observed that blocking the PI3K/Akt pathway contributed to restoring the podocyte adhesive capacity damage and autophagic activity [47]. In our present study, tetrandrine treatment reduced the levels of PI3K and the phosphorylated forms of Akt and increased the phosphorylation level of BAD. The observations of the present study suggest that the renoprotective effects of tetrandrine in MGN may be achieved via the PI3K/Akt pathway. However, it is important to consider that this study has some limitations. First, the hub targets obtained using the network pharmacological approach were not validated. Second, only one of the pathways was selected for validation in this study, which cannot fully explain the molecular mechanism of the pharmacological action of tetrandrine.

## 5. Conclusion

In summary, the pharmacological mechanism by which tetrandrine functions in the management of MGN was investigated using a combination of network pharmacology prediction and experimental validation. We demonstrate that tetrandrine can protect against MGN through the PI3K/Akt pathway. More importantly, these results suggest the use of tetrandrine as a potential therapeutic strategy for MGN treatment.

## Data Availability

The data used to support the findings of this study are available from the corresponding author upon request.

## Highlights

1. Tetrandrine has a variety of pharmacological effects.
2. We obtained 86 targets of tetrandrine for MGN treatment.
3. Tetrandrine treats MGN via P13 K/Akt signaling pathway in Heymann nephritis rat model.
